# Treatment pattern and outcomes of re-induction therapy prior to stem cell transplantation in patients with relapsed/refractory multiple myeloma in Germany

**DOI:** 10.1038/s41409-024-02208-3

**Published:** 2024-03-14

**Authors:** Sandra Sauer, Monika Engelhardt, Karolin Trautmann-Grill, Christoph Kimmich, Mathias Hänel, Martin Schmidt-Hieber, Hans Salwender, Carmen Flossmann, Hiltrud Heckmann, Franziska Ertel, Andrea Friederich, Sachin Patel, Barbara Thun, Marc S. Raab

**Affiliations:** 1 Department of Hematology, Oncology and Rheumatology, University Hospital Heidelberg, Heidelberg, Germany; 2 Medical Department, Hematology, Oncology & Stem Cell Transplantation, Faculty of Freiburg, Freiburg, Germany; 3 Medical Clinic I, University Hospital Carl Gustav Carus at the TU Dresden, Dresden, Germany; 4 Department of Oncology and Hematology, University Clinic Oldenburg, Oldenburg, Germany; 5 Department of Internal Medicine III, Chemnitz Hospital, Chemnitz, Germany; 6 Department of Hematology and Oncology, Carl-Thiem-Clinic, Cottbus, Germany; 7 Asklepios Tumorzentrum Hamburg, AK Altona and AK St Georg, Hamburg, Germany; 8 Amgen GmbH, Munich, Germany; 9 Amgen Ltd, Uxbridge, UK; 10 IQVIA Commercial GmbH & Co. OHG, Munich, Germany

**Keywords:** Cancer therapy, Combination drug therapy

## Abstract

There are limited data guiding choice of re-induction therapies for patients with relapsed/refractory multiple myeloma (RRMM) prior to stem cell transplantation (SCT). We performed a retrospective medical chart review of 171 patients with RRMM in Germany who received re-induction therapy in second line (78%; *n* = 134) or third line (22%; *n* = 37) prior to re-SCT. Index therapy was defined as first completed re-induction therapy for planned myeloablative conditioning and SCT in second/third line within the eligibility period (1/2016–12/2019). Most common pre-index first line and maintenance therapy used were bortezomib-based combinations (91%; *n* = 155/171) and lenalidomide (55%; *n* = 29/53), respectively. Median duration of index therapy line was 9 months; carfilzomib-based combinations were the most widely used in second/third line re-induction therapy (49%; *n* = 83/171), followed by daratumumab-based combinations (21%; *n* = 36/171). Overall response rates in second/third line were 87% after re-induction and 96% after SCT; median time to next treatment line after start of index therapy was 31 months; median progression-free survival (PFS) was 29 months; and median overall survival after index date was not reached. Based on these data, re-induction therapy with salvage SCT appears to be beneficial in selected patients with RRMM in clinical practice in Germany, translating into deep responses, long PFS and prolonged time to next treatment.

## Introduction

Recent advances in treatment approaches for multiple myeloma (MM) have improved patient survival, with a reported median overall survival (OS) of 7–10 years [1–7]. However, while most patients initially respond to treatment, almost all will experience relapse, and the disease may become refractory to formerly effective medication [8]. Many treatment options are available for patients with relapsed/refractory MM (RRMM) today, and treatment decisions are primarily based on efficacy and toxicity, including prior therapies and the associated duration of response [4, 9, 10]. Treatment with triplet or quadruplet combinations at each relapse is preferred (with ≥2 new drugs to which the patient is not refractory) [4, 8], and typical examples include bortezomib-based, carfilzomib-based and CD38-antibody–based therapy combinations [9, 10]. High-dose therapy (HDT, e.g. myeloablative conditioning) with autologous stem cell transplantation (ASCT) is a well-established first line treatment for MM; salvage HDT/ASCT should also be considered for eligible patients who relapse after first line treatment [9, 11]. Although data are limited, studies have demonstrated benefits of salvage ASCT after re-induction, including deep and durable responses and improved OS and progression-free survival (PFS) compared with other treatment options [11–13].

Regarding salvage stem cell transplantation (SCT), a key issue is the selection of an optimal re-induction therapy; nevertheless, guidance is limited, and the choice is often based on a patient’s response to, and toxicity of, prior therapies, their comorbidities and disease burden [14]. Furthermore, real-world evaluations of re-induction therapy prior to ASCT are scarce [15, 16], and there are currently no published real-world studies that focus on the German treatment landscape. More information regarding treatment outcomes and treatment sequencing could support decision-making for physicians and improve patient care.

The objective of this study was to describe patient characteristics, treatment pattern and treatment outcomes in patients with RRMM in Germany who had received re-induction therapy for planned SCT.

## Methods

### Study design

**Fig. 1:**
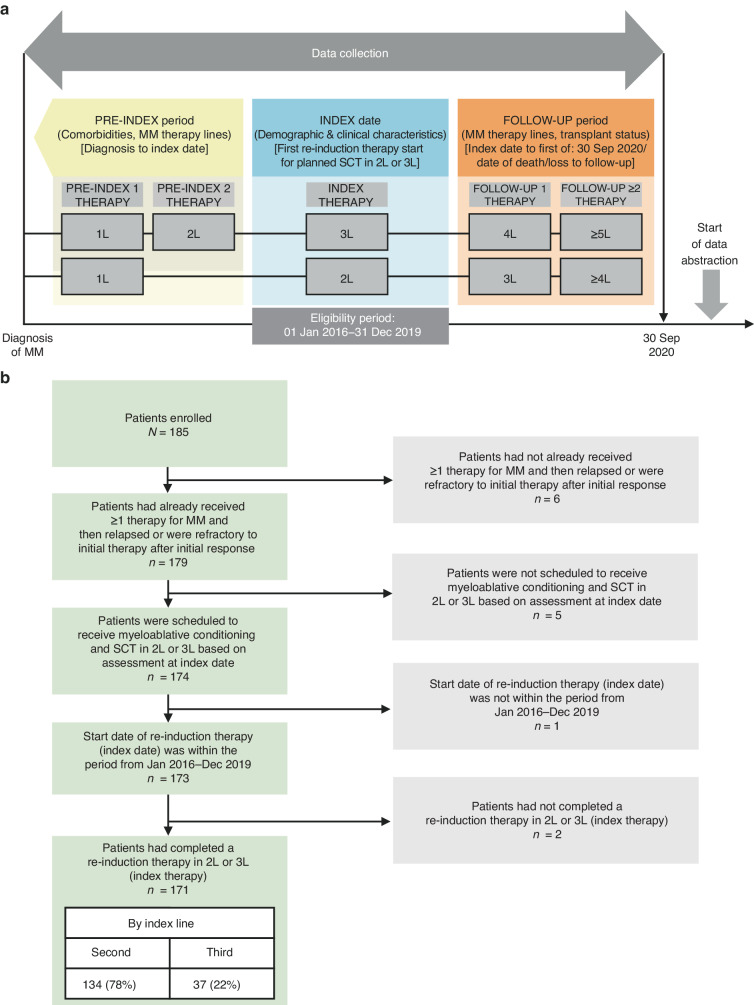
Study design and disposition of patients with relapsed/refractory multiple myeloma included in the analysis. **a** Study design and (**b**) patient disposition. 1L first line, 2L second line, 3L third line, 4L fourth line, 5L fifth line, MM multiple myeloma, SCT stem cell transplantation.

This was a retrospective, multicenter medical chart review study in patients with RRMM who had received re-induction therapy in second line or third line to facilitate SCT. The study design is depicted in Fig. 1a. Index therapy line was defined as the first completed re-induction therapy for planned SCT as well as follow-up therapy in second line or third line within the eligibility period (Jan 2016–Dec 2019); the start of this therapy was the index date. The pre-index period was between the diagnosis of MM and the index date, and the follow-up period ran from the index date until 30 Sep 2020, date of death or date of loss to follow-up (whichever occurred first).

The study planned to include >150 patients from ≥4 transplant centers in Germany. The sample size was chosen considering a confidence interval (CI) of 95% and a hypothetical proportion of 50%, which yields to the largest sample size for a precision of ≥8%. The study was approved by the respective local institutional review boards/independent ethics committees (listed in Supplementary Table 1) and was conducted in accordance with the Declaration of Helsinki and Good Clinical Practice guidelines. In addition to ethics approval, informed consent (via signed patient consent forms) was obtained from all patients by the respective study centers for data to be used for research purposes.

### Study population

Patients meeting the following criteria were eligible for inclusion in the study: aged ≥18 years at the index date; diagnosis of MM defined according to the International Myeloma Working Group (IMWG) criteria [17]; previously received ≥1 therapy for MM and relapsed or was refractory to the therapy after initial response; planned to receive myeloablative conditioning and SCT in second line or third line (based on assessment at index date); completed re-induction therapy in second line or third line; the index date was within the eligibility period.

### Study objectives and assessments

The primary objective was to describe patient characteristics and treatment pattern of patients with RRMM who had received re-induction therapy in second line or third line to facilitate SCT. Secondary objectives were to evaluate the proportion of patients who were transplanted after receiving re-induction therapy in second line or third line, the type of SCT (autologous single, autologous double [tandem], allogeneic, both autologous and allogeneic) and time to next treatment (TTNT). Exploratory objectives were to analyze the overall response rate (ORR), PFS and OS. Additional analyses were performed in patients stratified by index therapy line (second or third), maintenance therapy (yes or no) and therapy combination (carfilzomib-based or non-carfilzomib-based).

Full definitions for the evaluated treatment outcomes (TTNT, PFS and OS) are in the Supplementary methods. Treatment responses after re-induction therapy and after SCT were assessed according to the IMWG criteria [18].

Patient age, sex, weight and height, comorbidities, Eastern Cooperative Oncology Group performance status (ECOG-PS) and/or Karnofsky Index (within 14 days prior to index date) were collected at the index date. Date of MM diagnosis and International Staging System (ISS) stage and/or revised ISS at diagnosis were collected from the pre-index period. Data were collected on the type of anti-myeloma medication and duration of and the number of cycles for each line of therapy; the index date was collected for re-induction therapy.

### Statistical analyses

Analyses were performed using descriptive statistics. Continuous variables were described as medians and ranges. Categorical variables were summarized using frequency counts and percentages; 95% CIs were presented if applicable. Time-to-event analyses were performed using the Kaplan–Meier method.

## Results

### Baseline demographics and clinical characteristics

Data from medical charts of 171 patients were included in the analyses (Fig. 1b). The median age of patients was 61 years, 59% were male and 66% were overweight or obese (Table 1). Most patients had an ECOG-PS of 0 or 1 at the index date. More patients completed re-induction therapy in second line (78%, *n* = 134) than in third line (22%, *n* = 37).

**Table 1:** Baseline demographics and clinical characteristics at index date.

	Overall (*N* = 171)	Index therapy	
		2L (*n* = 134)	3L (*n* = 37)
Sex, *n* (%)			
Male	100 (58.5)	76 (56.7)	24 (64.9)
Age, years			
Median (range)	61.0 (32.0–73.0)	61.0 (42.0–73.0)	61.0 (32.0–69.0)
Age category, *n* (%)			
≤60 years	79 (46.2)	63 (47.0)	16 (43.2)
61–65 years	48 (28.1)	35 (26.1)	13 (35.1)
66–70 years	38 (22.2)	30 (22.4)	8 (21.6)
>70 years	6 (3.5)	6 (4.5)	0 (0.0)
BMI, kg/m^2^			
Median (range)	26.8 (17.5–45.5)	26.7 (17.3–45.5)	27.0 (20.0–41.3)
BMI category, *n* (%)			
Underweight (≤18.5 kg/m^2^)	1 (0.6)	1 (0.8)	0 (0.0)
Normal weight (18.5–24.9 kg/m^2^)	46 (26.9)	36 (26.9)	10 (27.0)
Overweight/obesity (≥25 kg/m^2^)	113 (66.1)	87 (64.9)	26 (70.3)
Missing data	11 (6.4)	10 (7.5)	1 (2.7)
ISS stage at first diagnosis, *n* (%)			
I	48 (28.1)	40 (29.9)	8 (21.6)
II	35 (20.5)	31 (23.1)	4 (10.8)
III	45 (26.3)	32 (23.9)	13 (35.1)
Missing data	43 (25.1)	31 (23.1)	12 (32.4)
Chronic comorbidities^a^, *n* (%)			
None	102 (59.6)	82 (61.2)	20 (54.1)
Any comorbidity	69 (40.4)	52 (38.8)	17 (45.9)
ECOG-PS^b^			
Median (range)	1.0 (0.0–2.0)	1.0 (0.0–2.0)	1.0 (1.0–2.0)
ECOG-PS single scale values, *n* (%)			
0–1	134 (78.4)	106 (79.1)	28 (75.6)
≥2	4 (2.3)	1 (0.7)	3 (8.1)
Missing data	33 (19.3)	27 (20.1)	6 (16.2)
Duration, months, median (range)			
Diagnosis of MM to end of study	79.1 (8.0–207.0)	78.0 (9.0–207.0)	85.0 (8.0–195.0)
Diagnosis of MM to index date^c^	46.0 (3.0–179.0)	45.0 (3.0–172.0)	47.0 (7.0–179.0)
Follow-up period^d^ since index date^c^	33.0 (1.0–56.5)	32.8 (2.9–56.5)	34.5 (1.0–56.5)

*2L* second line, *3L* third line, *BMI* body mass index, *ECOG-PS* Eastern Cooperative Oncology Group performance status, *ISS* International Staging System, *MM* multiple myeloma, *SCT* stem cell transplantation.

^a^Any malignancy (including leukemia and lymphoma), cerebrovascular disease, chronic liver disease (mild, moderate-to-severe), chronic pulmonary disease, dementia, diabetes mellitus with/without chronic complications, hemiplegia or paraplegia, peptic ulcer disease, peripheral vascular disease, metastatic solid tumor, myocardial infarction, renal disease, rheumatologic disease.

^b^Calculated based on ECOG-PS or converted Karnofsky Index.

^c^Index date: start of re-induction therapy for planned SCT in second line or third line.

^d^Follow-up period: period from start of index therapy line to either 30 Sep 2020, date of death or date of loss to follow-up (whichever occurred first).

### Treatment pattern

#### Pre-index first line therapy

**Fig. 2:**
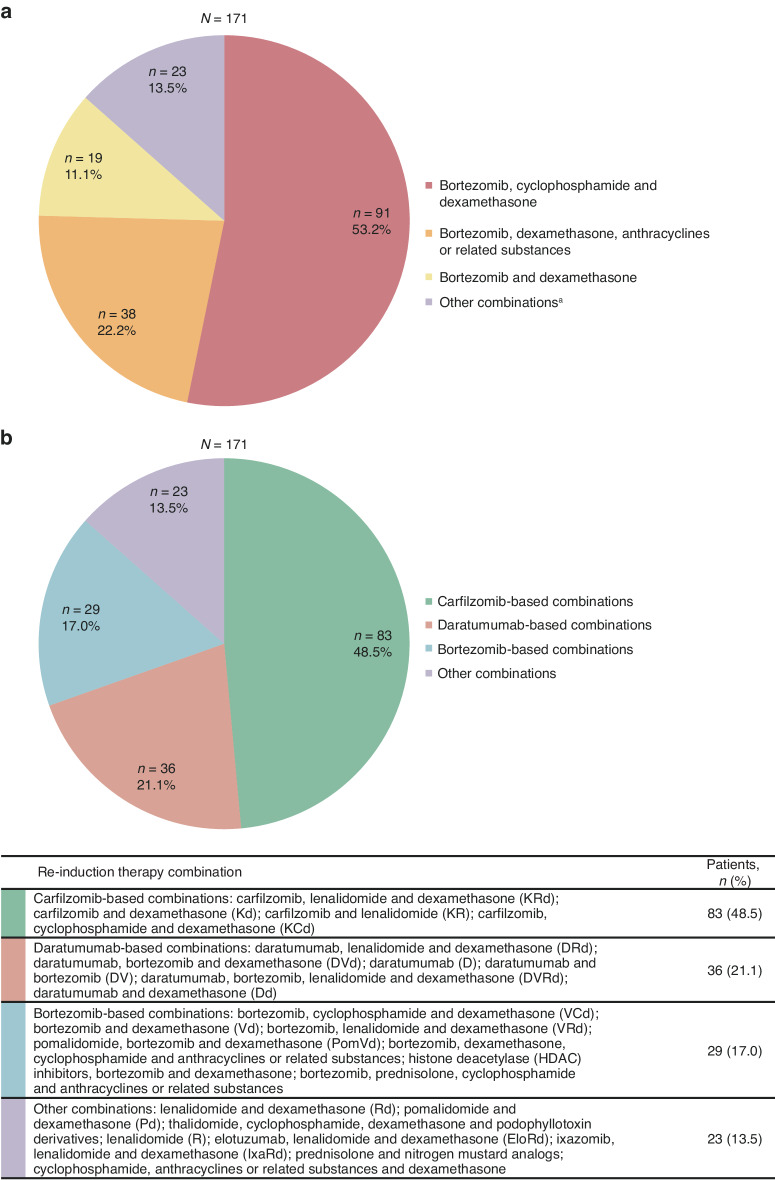
Anti-myeloma therapies received by patients with relapsed/refractory multiple myeloma. Anti-myeloma therapies received (**a**) as first line pre-index therapy and (**b**) as re-induction with second line or third line index therapy. *ATC* Anatomical Therapeutic Chemical. Owing to rounding, percentages may not add up to 100%. ^a^Combinations used in <5 patients are summarized under “Other combinations”: bortezomib and cyclophosphamide; bortezomib and lenalidomide; bortezomib, lenalidomide and dexamethasone; bortezomib, melphalan and prednisolone; elotuzumab, bortezomib, lenalidomide and dexamethasone; lenalidomide and dexamethasone; lenalidomide, anthracyclines or related substances (ATC code: L01DB) and dexamethasone; thalidomide, anthracyclines or related substances (ATC code: L01DB) and dexamethasone; anthracyclines or related substances (ATC code: L01DB) and dexamethasone; cyclophosphamide, anthracyclines or related substances (ATC code: L01DB) and dexamethasone; cyclophosphamide, podophyllotoxin derivatives and dexamethasone; nitrogen mustard analogs, and prednisolone; and vinca alkaloids and analogs, anthracyclines or related substances (ATC code: L01DB) and dexamethasone.

Bortezomib-based combinations were used as first line anti-myeloma therapy in 91% of patients, of which, bortezomib, cyclophosphamide and dexamethasone (VCd) was the most widely used (53% of patients) (Fig. 2a and Supplementary Table 2). Most (88%, *n* = 150) patients received HDT/SCT as first line therapy; 79% of these patients had single ASCT and 21% had tandem ASCTs (Supplementary Table 3).

In total, 11% (*n* = 19) of patients received consolidation and nearly one-third (31%, *n* = 53) received first line maintenance (Supplementary Table 2). Lenalidomide was most frequently used for maintenance (55%, *n* = 29).

Results by index therapy line (2L/3L) are presented in Supplementary Tables 2 and 3. The duration of first line therapy in all patients, including those with or without maintenance therapy, is presented in Supplementary Table 4.

#### Index therapy line (second or third line therapy applied)

Carfilzomib-based combinations were the most widely used (49%) re-induction therapy overall, followed by daratumumab-based (21%), bortezomib-based (17%) and other (13%) combinations (Fig. 2b). Carfilzomib, lenalidomide and dexamethasone (KRd) was the most common combination in second line (48%) and third line (22%), followed by daratumumab, lenalidomide and dexamethasone (DRd; 13% and 14%, respectively) (Supplementary Table 5). The median (range) number of cycles for re-induction therapy was 3 (1–19). In patients who received carfilzomib-based (*n* = 83) versus non-carfilzomib-based (*n* = 88) combinations (Fig. 2b), the median (range) number of cycles was similar with 3 (1–12) and 4 (2–19), respectively. All patients received HDT/SCT in second/third line: 85% received single ASCT, the remaining patients received either allogeneic SCT (10%), ASCT plus allogeneic SCT (4%) or tandem ASCT (1%) (Supplementary Table 3). More than half (54%) of patients received maintenance at second line (62%) and third line (24%); in most cases, patients received lenalidomide monotherapy (80%) for maintenance (Supplementary Table 5). In the carfilzomib-based and non-carfilzomib-based combinations subgroups, respectively, 52% and 56% of patients received maintenance; again, with the majority (88% and 73%) receiving lenalidomide monotherapy.

The duration of index therapy in all patients, by index therapy line and in patients with or without maintenance therapy, is presented in Supplementary Table 4. The median (range) duration of re-induction therapy was 3 (0.9–14.7) months, whereas the median duration of index therapy line was 9 (1.0–44.0) months. In the carfilzomib-based combinations subgroup, the median (range) duration of index therapy line in patients without or with maintenance therapy was 5 (1.0–13.0) months and 22 (8.0–44.0) months, respectively; in the non-carfilzomib-based combinations subgroup, this was 6 (1.0–17.0) months and 20 (5.0–43.0) months, respectively.

### Treatment outcomes

#### Overall response rates

**Fig. 3:**
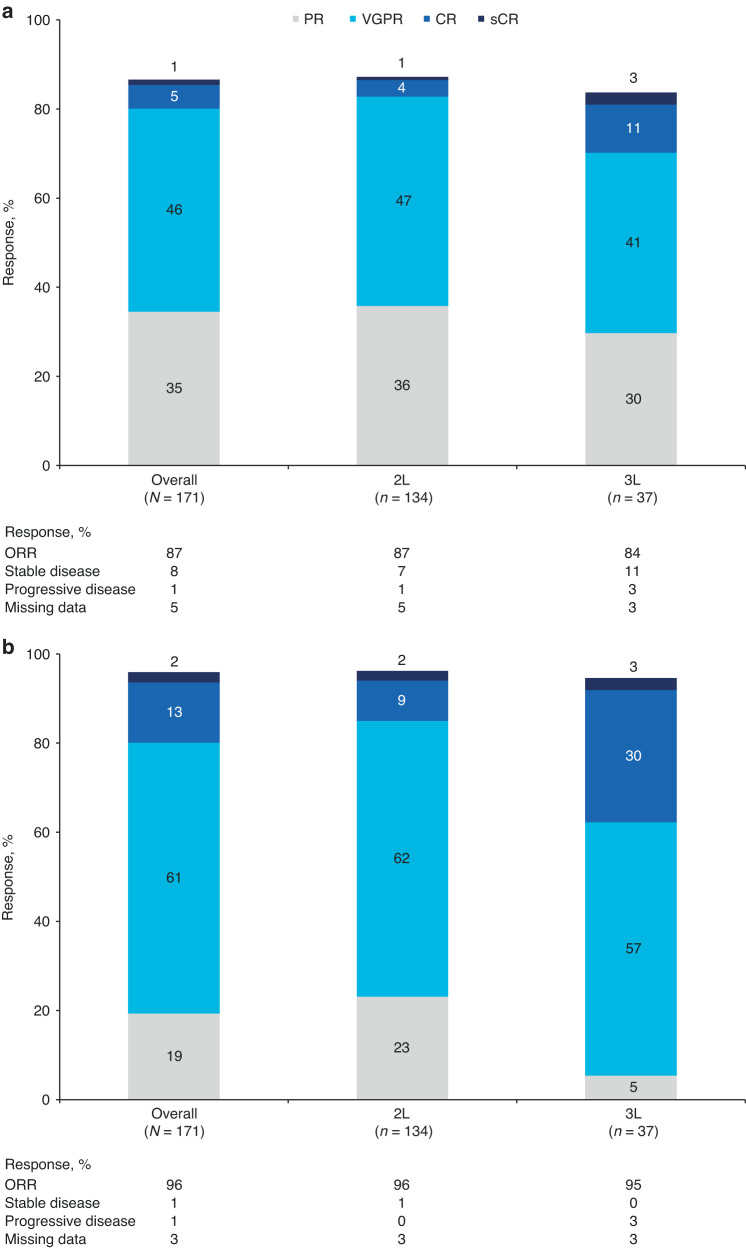
Overall response rates^a,b^ for index therapy overall and by index therapy line (second line or third line) in patients with relapsed/refractory multiple myeloma. Overall response rates **a** after re-induction and **b** after stem cell transplantation in second line or third line. 2L second line, 3L third line, CR complete response, IMWG International Myeloma Working Group, ORR overall response rate, PR partial response, sCR stringent CR, VGPR very good partial response. ^a^Using IMWG criteria for multiple myeloma [18]; ^b^Percentages may not add up to 100% due to rounding.

High ORRs during index therapy were observed in patients after re-induction (87%; 95% CI: 80.5, 91.3) and SCT (96%; 95% CI: 91.8, 98.3) (Fig. 3). A very good partial response (VGPR) or better was achieved in 52% of patients after re-induction therapy and in 77% of patients after SCT.

In second line and third line index therapy, respectively, the ORR (95% CI) was 87% (80.5, 92.4) and 84% (68.0, 93.8) after re-induction therapy, and 96% (91.5, 98.8) and 95% (81.8, 99.3) after SCT. Correspondingly, a VGPR or better was achieved in 51% and 54% of patients after re-induction therapy and in 73% and 89% of patients after SCT, respectively (Fig. 3).

**Fig. 4:**
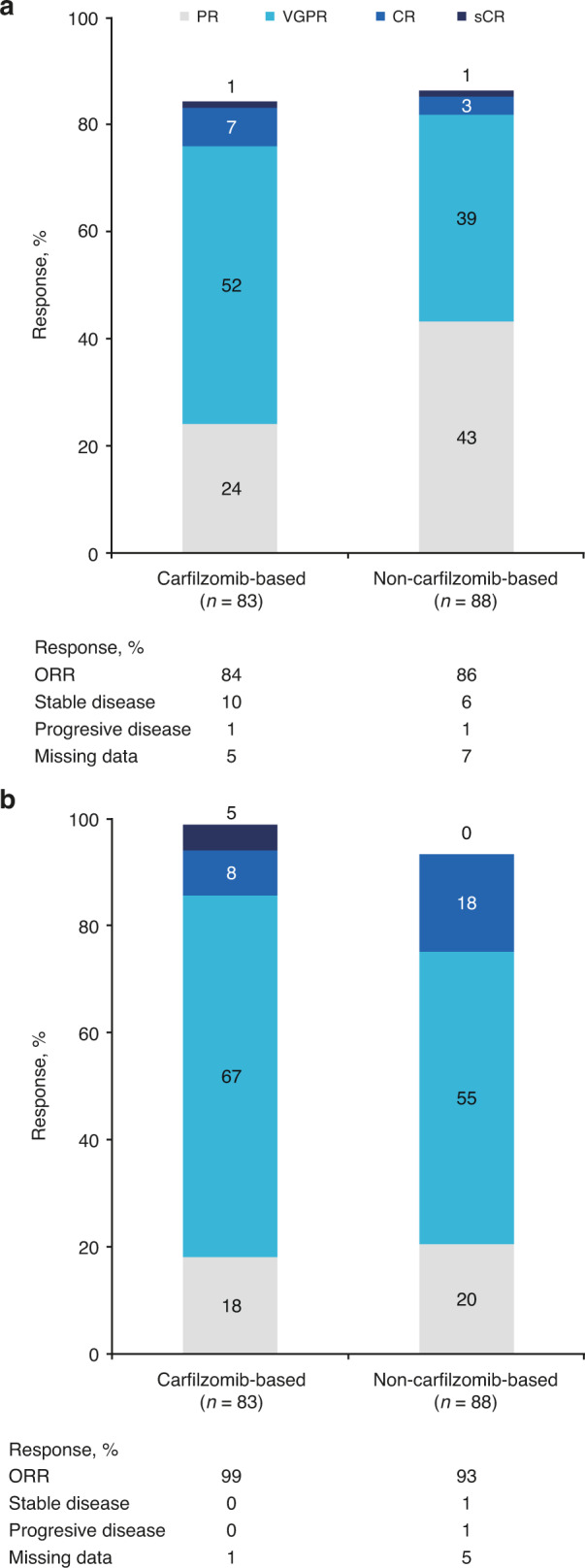
Overall response rates^a,b^ for index therapy (second line and third line) by carfilzomib-based or non-carfilzomib-based index therapy combinations in patients with relapsed/refractory multiple myeloma. Overall response rates (**a**) after re-induction and (**b**) after stem cell transplantation. CR complete response, IMWG International Myeloma Working Group, ORR overall response rate, PR partial response, sCR, stringent CR, VGPR very good partial response. ^a^Using IMWG criteria for multiple myeloma [18]; ^b^Percentages may not add up to 100% due to rounding.

ORRs (95% CI) in the carfilzomib-based and non-carfilzomib-based combinations subgroups, respectively, were 84% (74.7, 91.4) and 86% (77.4, 92.8) after re-induction therapy, and 99% (93.5, 100.0) and 93% (85.8, 97.5) after SCT. Correspondingly, a VGPR or better was achieved in 60% and 43% of patients after re-induction therapy and in 81% and 73% of patients after SCT, respectively (Fig. 4).

#### TTNT, PFS and OS

**Fig. 5:**
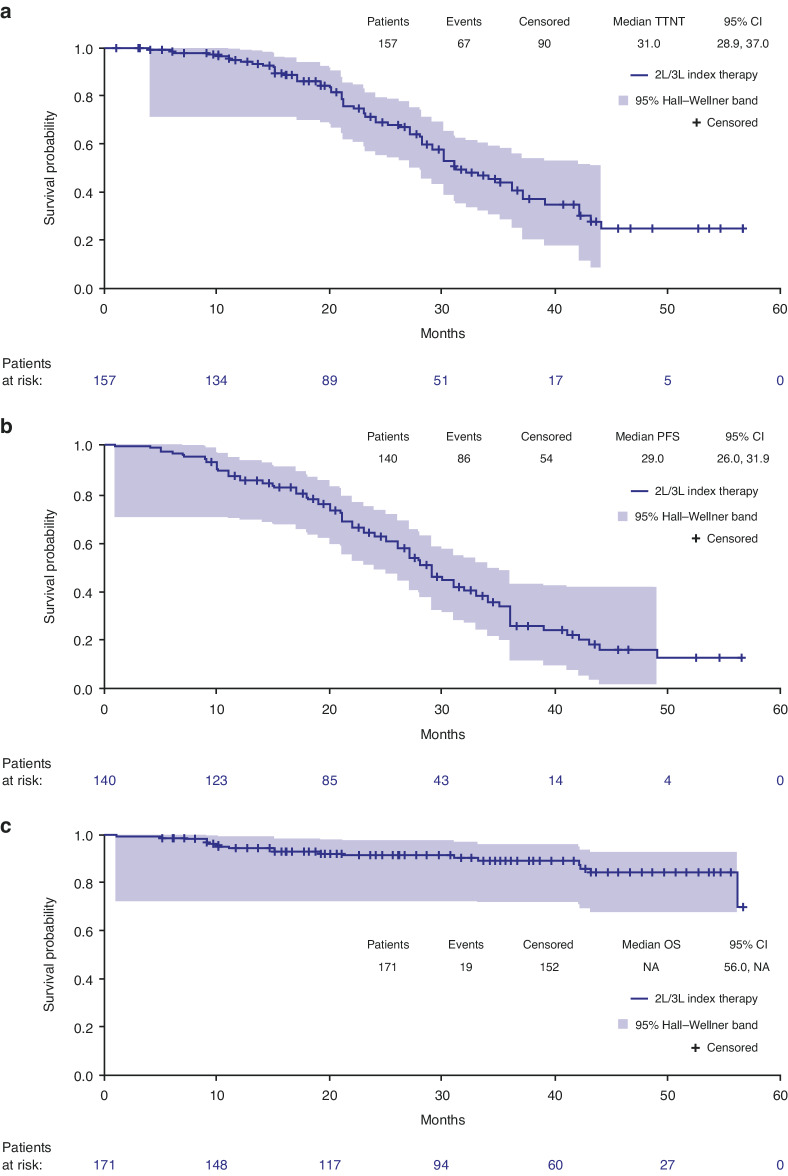
Treatment outcomes in index therapy (2L/3L), in patients with relapsed/refractory multiple myeloma. **a** Time to next treatment^a^, **b** progression-free survival^b^ and (**c**) overall survival^c^. *2L* second line, *3L* third line, CI confidence interval, IMWG International Myeloma Working Group, NA not applicable, PFS progression-free survival, TTNT time to next treatment. ^a^Time from the start of index therapy line to the start of next follow-up therapy line. Data from patients with no follow-up therapy lines were censored on 30 Sep 2020, date of death or date of loss to follow-up (whichever occurred first); ^b^Time from the start of index therapy line to disease progression (as defined by the IMWG criteria [18]), death or start of new therapy line. Data from surviving patients without progression who were not documented as lost to follow-up were censored (for the index line) on 30 Sep 2020 if no follow-up therapy lines were documented. Patients with a new therapy line were excluded from the analysis if the start date of the new therapy line was unknown; ^c^Time from the start of index therapy line until end of observation period (30 Sep 2020) or death. Patients who were alive at end of observation period (30 Sep 2020) were right censored to the end of observation period. Observation period: from the start of index therapy line to 30 Sep 2020 or death or date of loss to follow-up (whichever occurred first).

Median treatment outcomes (TTNT, PFS and OS) were analyzed from the start of index therapy line. Median TTNT was 31 months (2.6 years; 95% CI: 28.9, 37.0 months) (Fig. 5a), and median PFS was 29 months (2.4 years; 95% CI: 26.0, 31.9 months) (Fig. 5b). Median OS was not reached during the median observation period of 33 (range 1.0–56.5) months; however, the survival probability was 84% after 50 months (4.2 years) (Fig. 5c).

In second line and third line index therapy, median TTNT was 34 months (2.8 years; 95% CI: 30.0, 42.0) and 25 months (2.1 years; 95% CI: 21.0, 28.9) (Supplementary Fig. 1a), respectively, and median PFS was 31 months (2.6 years; 95% CI: 27.0, 34.0) and 22 months (1.8 years; 95% CI: 15.0, 28.0) (Supplementary Fig. 1b). Median OS from the start of index therapy line was not reached during the median observation period of 33 (range 2.9–56.5) months for second line index therapy and 35 (range 1.0–56.5) months for third line index therapy; the survival probability was 88% and 72%, respectively, after 50 months (4.2 years) (Supplementary Fig. 1c).

In patients with or without maintenance therapy in index line, the median TTNT was 36 months (3.0 years; 95% CI: 30.0, not applicable) and 28 months (2.3 years; 95% CI: 22.0, 35.0), respectively (Supplementary Fig. 2). The median PFS was comparable for the carfilzomib-based and non-carfilzomib-based index therapy combinations subgroups (Supplementary Fig. 3).

## Discussion

In this retrospective study of 171 patients with RRMM in Germany, high ORRs were observed after re-induction therapy and SCT, irrespective of therapy line (second or third) or combination therapy (carfilzomib-based or non-carfilzomib-based combinations). High rates of VGPR or better were observed in the overall patient population after re-induction (mainly with carfilzomib-based and daratumumab-based regimens), as well as after salvage SCT. These re-induction combinations, commonly used in RRMM, led to long TTNT, PFS and OS. Patients receiving maintenance therapy after salvage SCT benefited from a prolonged duration of index therapy and TTNT compared with those without maintenance therapy. Overall, these data emphasize that re-induction and salvage SCT remain valid and effective treatment options in RRMM, despite the emergence of new myeloma therapies, especially if the patient’s peripheral blood stem cells are still available after mobilization in first line therapy and a second ASCT can be performed rather quickly. In Germany, induction followed by HDT/ASCT is recommended for patients aged <70 years [9, 19]; therefore, the median age of 61 years in our overall study population is consistent with typical eligibility for ASCT. A substantial proportion of patients in our study (66%) had a body mass index of ≥25.0 kg/m^2^, which is representative of the German population in 2019–2020 [20].

Regarding pre-index first line therapy, the majority (91%) of patients received bortezomib-based therapy combinations and most (88%) received SCT. Given that induction therapy with triplets and quadruplets is recommended for transplant-eligible patients with MM [9], the observed treatment pattern was as expected. More than half of patients received VCd at first line; this induction combination was widely used in Germany at the time of data collection, prior to the quadruplet daratumumab, bortezomib, thalidomide and dexamethasone (DVTd) becoming the new standard of care following its approval in 2019 [9, 21].

There are multiple recommended options for second line combination therapies for RRMM, depending on the context of clinical relapse, such as prior therapy and refractoriness to lenalidomide, duration of response and eligibility for salvage ASCT [9, 10]. Salvage ASCT should be considered in patients who did not receive first line ASCT or who responded well to first line treatment, including ASCT with maintenance therapy, and did not experience disease progression within 2–3 years [9, 10]. Limited guidance is available regarding optimal re-induction combinations for RRMM, particularly for patients who have received ≥2 previous therapy lines [9, 10]. In the present study, carfilzomib-based combinations were most frequently used for re-induction (49%); KRd was the most common in both second line and third line (48% and 22%, respectively), reflecting the fitness of patients deemed eligible for repeat SCT. In another retrospective chart review of patients with RRMM in Germany in 2017 and 2018, patients most frequently received either daratumumab-based or carfilzomib-based therapies in second line [22], confirming the importance of these potent combination partners. In our study, the standard duration of re-induction before re-transplantation was a median of 3 cycles, which is comparable with other published data [11, 13].

The low rate of consolidation therapy at first line and index therapy (11% and 1%, respectively) is not surprising. Although published studies support a role for consolidation therapy [23, 24], its use post-ASCT has not been established as a standard procedure in Germany prior to the approval of DVTd [9, 21].

After first line ASCT, maintenance therapy is indicated to prolong remission, with lenalidomide as the standard of care [9, 25]. In our study, 31% of patients received maintenance at first line and over half (54%) at index line after SCT, most commonly lenalidomide monotherapy (55% and 80%, respectively). The median duration of first line maintenance was 23 months (with a broad range of 2–160 months). After approval by the European Medicines Agency (EMA) as first line maintenance therapy in September 2018 [26], continued lenalidomide therapy after salvage ASCT now represents standard practice in Germany. Although lenalidomide-refractoriness is relevant to treatment decisions in RRMM [27], it was not assessed in this chart review. The effect of lenalidomide pre-treatment on our patient population was expected to be minor as data collection of re-induction therapy mostly took place prior to the approval of lenalidomide maintenance therapy by the EMA.

Patients responded very well to re-induction and SCT (high ORRs of 87% and 96%, respectively), and treatment outcomes in subgroups receiving carfilzomib-based or non-carfilzomib-based index therapy combinations were generally similar. In an analysis of the randomized Phase 3 study ASPIRE in patients with RRMM, the ORR after KRd therapy in patients with late relapse (after >1 year) was 89% [28]. Other clinical and observational studies have reported ORRs of 84–95% in patients with RRMM after carfilzomib-based or daratumumab-based combination therapy in second line or third line [22, 28–30], and of 96–100% after bortezomib-based or daratumumab-based combination re-induction therapy and salvage ASCT [15, 16]. Our results demonstrated that after re-induction and SCT, 6% and 16% of patients, respectively, achieved a CR or better, and 52% and 77% had a VGPR or better (with most patients treated with KRd as re-induction). After re-induction (with mainly daratumumab, carfilzomib, lenalidomide and dexamethasone [DKRd] or carfilzomib-based, bortezomib-based or daratumumab-based combinations) and salvage HDT/ASCT, previous studies reported CR or better in 23–67% of patients and VGPR or better in 71–78% of patients [15, 16, 31, 32]. The apparently low rates of stringent CR and CR in the present study may reflect underreporting, as bone marrow punctures are not routinely performed after re-induction and after SCT outside of clinical trials, and data for this were not collected. Of note, the present study assessed re-induction as well as salvage SCT in daily clinical practice. As the focus of our study was on re-induction therapies for re-transplantation, patients with RRMM without transplantation were not included in this analysis. Previously, a real-world study reported VGPR or better in 64% of patients who received second line treatment; 95% of these patients received non-transplant therapies [22]. Another real-world study reported VGPR or better, by line of therapy: in second line, in 71% of patients who received KRd and 59% of patients who received the doublet carfilzomib and dexamethasone (Kd); in third line, in 68% and 49% of KRd and Kd patients, respectively [33]. These results are comparable with response rates in patients after re-induction reported in our study.

The median TTNT of 31 months reported in our study is a noteworthy result in the setting of RRMM, supported by the median PFS of 29 months both in the overall population and when stratified by index therapy type. This is comparable to other recent retrospective studies of salvage SCT that reported a median PFS of 23–33 months after re-induction with KRd and HDT/ASCT, or salvage ASCT [11, 34, 35].

The OS data are also encouraging, with a survival probability of 84% at 4.2 years from the index date. The benefits of salvage ASCT on OS and PFS are further supported by a small number of randomized Phase 3 trials that reported improvements in OS and PFS after salvage ASCT and after re-induction therapy and HDT/ASCT, when compared with conventional therapy [12, 13].

As with all retrospective studies, a limitation of the present analysis was the collection of restrictive data from medical records. Additionally, data were only collected until 30 Sep 2020; hence, treatment outcomes or discontinuations after this date were entered as missing. As data collection was anonymized, manual queries or source data verification could not be performed, and data were analyzed as reported by the sites. By selecting patients with a relapse-free period of ≥2–3 years after a first SCT, this study included a fit population of patients with MM. Within this study, data for patients with planned but not performed SCT were not documented. Furthermore, OS data may be affected by selection bias, as our study only included patients with a fitness status that allowed for consideration of SCT.

In conclusion, our data reflect clinical practice in patients with RRMM in Germany and demonstrate the benefits of salvage SCT after re-induction therapy, mainly with carfilzomib-based combinations. A selected group of mostly fit patients aged <70 years who responded well to first line therapy, with or without SCT, appeared to benefit from re-induction and SCT in second line and third line therapy. Although lenalidomide maintenance is only approved for first line therapy, in clinical practice, it may also be beneficial in later lines when patients are still sensitive to immunomodulatory drugs. The recently approved combinations for second line therapy (e.g. carfilzomib, daratumumab and dexamethasone, or isatuximab, carfilzomib and dexamethasone) now offer additional options for relapsed MM therapy [36]. New immunomodulatory cereblon E3 ligase modulators (e.g. iberdomide or mezigdomide), as well as chimeric antigen receptor T-cell therapies and bispecific antibodies, are under evaluation and are either already used or will be available for lenalidomide-refractory patients in the earlier RRMM setting [36]. Overall, the number of options for re-induction therapy is therefore increasing, and novel real-world data remain valuable for evaluating their impact on patient care.

### Supplementary information

Supplementary materials

## Data Availability

The datasets generated and/or analyzed during the current study are available from the corresponding author on reasonable request. The data that support the findings of this study are available from the study sponsor Amgen (GmbH) upon reasonable request. Qualified researchers may request data from Amgen studies. Complete details are available at http://www.amgen.com/datasharing.
