# Supplementary material for: Treatment pattern and outcomes of re-induction therapy prior to stem cell transplantation in patients with relapsed/refractory multiple myeloma in Germany
Source: Bone Marrow Transplant. 2024 Mar 14;59(6):880–9. doi: 10.1038/s41409-024-02208-3 (PMC11161411; doi:10.1038/s41409-024-02208-3)
Supplement: Supplementary file 1 — Supplementary materials [file 41409_2024_2208_MOESM1_ESM.pdf]

Supplementary materials

Supplementary methods

Study assessments – treatment outcome definitions

Time to next treatment was defined as the interval from the start of index therapy line to the start of next follow-up therapy line; data from patients with no follow-up therapy lines were censored on 30 Sep 2020, date of death or date of loss to follow-up (whichever occurred first). Progression-free survival was defined as the time from the start of index therapy line to disease progression (defined by the International Myeloma Working Group criteria [1]), death or start of new therapy line; data from surviving patients without progression who were not documented as lost to follow-up were censored (for the index line) on 30 Sep 2020 if no follow-up therapy lines were documented. Patients with a new therapy line were excluded from the analysis if the start date of the new therapy line was unknown. Overall survival was defined as the time from the start of index therapy line to the end of the follow-up period or death of any cause; surviving patients at the end of the follow-up period were censored on 30 Sep 2020 or date of loss to follow-up.

Supplementary tables

Supplementary Table 1 Ethics committees.

Ethics committee name	Reference number
Ethikkommission der Medizinischen Fakultät Heidelberg	S-232/2021
Ethikkommission an der TU Dresden	BO-EK-375072021
Ethikkommission der Landesärztekammer Brandenburg	2021-2110-BO
Ethik-Kommission der Universitätsklinik Freiburg	21-1511
Ethik-Kommission der Ärztekammer Hamburg	2021-200021-BO-bet
Medizinische Ethikkommission an der Carl von Ossietzky Universität Oldenburg	2021-104
Ethikkommission der Sächsischen Landesärztekammer	EK-BR-56/21-1

Supplementary Table 2 First-line anti-myeloma therapy, maintenance therapy and consolidation therapy, overall and by index therapy (2L/3L).

	Overall (*N* = 171)	Index therapy	
		2L (*n* = 134)	3L (*n* = 37)
**First-line drug combinations, *n* (%)**			
Bortezomib, cyclophosphamide and dexamethasone (VCd)	91 (53.2)	79 (59.0)	12 (32.4)
Bortezomib, anthracyclines or related substances[a] and dexamethasone	38 (22.2)	23 (17.2)	15 (40.5)
Bortezomib and dexamethasone (Vd)	19 (11.1)	14 (10.4)	5 (13.5)
Other combinations[b]	23 (13.5)	18 (13.4)	5 (13.5)
**Consolidation therapy, *n* (%)**			
Yes	19 (11.1)	17 (12.7)	2 (5.4)
No	130 (76.0)	108 (80.6)	22 (59.5)
Missing data	22 (12.9)	9 (6.7)	13 (35.1)
**Treatment used**			
Valid, *N* (%)[c]	19 (100)	17 (100)	2 (100)
Bortezomib and lenalidomide (VR), *n* (%)	1 (5.3)	1 (5.9)	0 (0.0)
Lenalidomide (R), *n* (%)	18 (94.7)	16 (94.1)	2 (100.0)
**Maintenance therapy, *n* (%)**			
Yes	53 (31.0)	41 (30.6)	12 (32.4)
No	117 (68.4)	92 (68.7)	25 (67.6)
Missing data	1 (0.6)	1 (0.7)	0 (0.0)
**Treatment used**			
Valid, *N* (%)[d]	53 (100)	41 (100)	12 (100)
Lenalidomide (R), *n* (%)	29 (54.7)	24 (58.5)	5 (41.7)
Thalidomide (T), *n* (%)	12 (22.6)	9 (22.0)	3 (25.0)
Other drugs[e], *n* (%)	12 (22.6)	8 (19.5)	4 (33.3)

2L second-line, *3L* third-line, *ATC* Anatomical Therapeutic Chemical.
[a]ATC code: L01DB; [b]Combinations used in <5 patients, including anthracyclines or related substances (ATC code: L01DB; e.g. doxorubicin) and dexamethasone; bortezomib and cyclophosphamide; bortezomib and lenalidomide; bortezomib, lenalidomide and dexamethasone; bortezomib, melphalan and prednisolone; cyclophosphamide, anthracyclines and related substances (ATC code: L01DB) and dexamethasone; cyclophosphamide, podophyllotoxin derivatives (e.g. etoposide) and dexamethasone; elotuzumab, bortezomib, lenalidomide and dexamethasone; lenalidomide and dexamethasone; lenalidomide, anthracyclines or related substances (ATC code: L01DB) and dexamethasone; nitrogen mustard analogs (e.g. bendamustin) and prednisolone; thalidomide, anthracyclines or related substances (ATC code: L01DB) and dexamethasone; and vinca alkaloids and analogs (e.g. vincristine), anthracyclines or related substances (ATC code: L01DB) and dexamethasone; [c]Number of patients with consolidation therapy = “Yes”; [d]Number of patients with maintenance therapy = “Yes”; [e]Combinations/drugs used in <10 patients, including bortezomib, interferon, ixazomib, pegintron and experimental drugs not yet approved.

Supplementary Table 3 Stem cell mobilization/HDT/SCT in first-line and after re-induction in second-line or third-line index therapy, overall and by index therapy (2L/3L).

	**Overall (*N* = 171)**	**Index therapy**	
		**2L (*n* = 134)**	**3L (*n* = 37)**
**1L**			
Stem cell mobilization, *n* (%)			
Yes	155 (90.6)	127 (94.8)	28 (75.7)
No	16 (9.4)	7 (5.2)	9 (24.3)
HDT and SCT, *n* (%)			
Yes	150 (87.7)	126 (94.0)	24 (64.9)
No	5 (2.9)	1 (0.7)	4 (10.8)
Missing data	16 (9.4)	7 (5.2)	9 (24.3)
Type of SCT			
Valid, *N* (%)[a]	150 (100)	126 (100)	24 (100)
Autologous single, *n* (%)	119 (79.3)	102 (81.0)	17 (70.8)
Autologous double (tandem), *n* (%)	31 (20.7)	24 (19.0)	7 (29.2)
Allogeneic, *n* (%)	0 (0.0)	0 (0.0)	0 (0.0)
Autologous and allogeneic, *n* (%)	0 (0.0)	0 (0.0)	0 (0.0)
**2L or 3L**			
Stem cell mobilization, *n* (%)			
Yes	21 (12.3)	18 (13.4)	3 (8.1)
No	149 (87.1)	116 (86.6)	33 (89.2)
Missing data	1 (0.6)	0 (0.0)	1 (2.7)
HDT and SCT, *n* (%)			
Yes	171 (100.0)	134 (100.0)	37 (100.0)
No	0 (0.0)	0 (0.0)	0 (0.0)
Type of SCT, *n* (%)			
Autologous single	146 (85.4)	122 (91.0)	24 (64.9)
Autologous double (tandem)	1 (0.6)	1 (0.8)	0 (0.0)
Allogeneic	17 (9.9)	5 (3.7)	12 (32.4)
Autologous and allogeneic	7 (4.1)	6 (4.5)	1 (2.7)

1L first-line, *2L* second-line, *3L* third-line, *HDT* high-dose therapy, *SCT* stem cell transplantation.
[a]Number of patients with SCT = “Yes”.

Supplementary Table 4 Duration of first-line, index and maintenance therapy, overall, by index therapy (2L/3L) and by maintenance therapy.

	Overall	Index therapy		Maintenance therapy	
		2L	3L	Yes	No
**Duration of 1L therapy[a], months**					
Total, *N*	171	134	37	53	117
Valid, *N* (%)[b]	170 (99.4)	133 (99.3)	37 (100)	53 (100)	117 (100)
Median (range)	6.1 (1.0–168.0)	6.1 (1.9–168.0)	7.0 (1.0–63.0)	33.0 (10.0–168.0)	5.0 (1.0–12.0)
Missing data, *N* (%)	1 (0.6)	1 (0.7)	0	0	0
**Duration of maintenance therapy at 1L, months**					
Total, *N*	53	41	12	53	–
Valid, *N* (%)[b]	53 (100)	41 (100)	12 (100)	53 (100)	–
Median (range)	23.0 (2.0–160.0)	23.0 (2.0–160.0)	13.5 (3.0–48.0)	23.0 (2.0–160.0)	–
Missing data, *N* (%)	0	0	0	0	–
**Duration of index therapy[c], months**					
Total, *N*	171	134	37	92	68
Valid, *N* (%)[b]	113 (66.1)	80 (59.7)	33 (89.2)	45 (48.9)	68 (100)
Median (range)	9.0 (1.0−44.0)	10.0 (3.0−44.0)	6.0 (1.0−40.0)	21.0 (5.0–44.0)	6.0 (1.0–17.0)
Missing data, *N* (%)	58 (33.9)	54 (40.3)	4 (10.8)	47 (51.1)	0
**Duration of re-induction therapy, months**					
Total, *N*	171	134	37	–	–
Valid, *N* (%)[b]	129 (75.4)	114 (85.1)	15 (40.5)	–	–
Median (range)	2.8 (0.9–14.7)	2.8 (1.8–14.7)	3.7 (0.9–8.4)	–	–
Missing data, *N* (%)	42 (24.6)	20 (14.9)	22 (59.5)	–	–
**Duration of maintenance therapy at index line, months**					
Total, *N*	92	83	9	92	–
Valid, *N* (%)[b]	48 (52.2)	43 (51.8)	5 (55.6)	48 (52.2)	–
Median (range)	12.0 (1.0–33.0)	12.0 (1.0–33.0)	8.0 (5.0–21.0)	12.0 (1.0–33.0)	–
Missing data, *N* (%)	44 (47.8)	40 (48.2)	4 (44.4)	44 (47.8)	–

1L first-line, *2L* second-line, *3L* third-line.

[a]Of all documented treatment schemes for first-line therapy; [b]Observations with non-missing values; [c]Of all documented treatment schemes for re-induction therapy.

Supplementary Table 5 Re-induction therapy, maintenance therapy and consolidation therapy overall, and by index therapy (2L/3L).

	**Overall (*N* = 171)**	**Index therapy**	
		**2L (*n* = 134)**	**3L (*n* = 37)**
**Re-induction therapy used[a], *n* (%)**			
Carfilzomib, lenalidomide and dexamethasone (KRd)	72 (42.1)	64 (47.8)	8 (21.6)
Daratumumab, lenalidomide and dexamethasone (DRd)	23 (13.5)	18 (13.4)	5 (13.5)
Bortezomib, cyclophosphamide and dexamethasone (VCd)	16 (9.4)	15 (11.2)	1 (2.7)
Lenalidomide and dexamethasone (Rd)	10 (5.8)	9 (6.7)	1 (2.7)
Carfilzomib and dexamethasone (Kd)	8 (4.7)	3 (2.2)	5 (13.5)
Daratumumab, bortezomib and dexamethasone (DVd)	7 (4.1)	6 (4.5)	1 (2.7)
Bortezomib and dexamethasone (Vd)	4 (2.3)	4 (3.0)	0 (0.0)
Bortezomib, lenalidomide and dexamethasone (VRd)	3 (1.8)	2 (1.5)	1 (2.7)
Daratumumab (D)	3 (1.8)	1 (0.7)	2 (5.4)
Pomalidomide and dexamethasone (Pomd)	3 (1.8)	0 (0.0)	3 (8.1)
Thalidomide, cyclophosphamide, podophyllotoxin derivatives and dexamethasone	3 (1.8)	1 (0.7)	2 (5.4)
Bortezomib, cyclophosphamide, anthracyclines or related substances[b] and dexamethasone	2 (1.2)	0 (0.0)	2 (5.4)
Pomalidomide, bortezomib and dexamethasone (PomVd)	2 (1.2)	2 (1.5)	0 (0.0)
Carfilzomib and lenalidomide (KR)	2 (1.2)	2 (1.5)	0 (0.0)
Lenalidomide (R)	2 (1.2)	2 (1.5)	0 (0.0)
Elotuzumab, lenalidomide and dexamethasone (EloRd)	2 (1.2)	2 (1.5)	0 (0.0)
Daratumumab and bortezomib (DV)	1 (0.6)	1 (0.7)	0 (0.0)
Bortezomib, histone deacetylase (HDAC) inhibitors and dexamethasone	1 (0.6)	0 (0.0)	1 (2.7)
Daratumumab, bortezomib, lenalidomide and dexamethasone (DVRd)	1 (0.6)	1 (0.7)	0 (0.0)

Bortezomib, prednisolone, cyclophosphamide and anthracyclines or related substances[b]	1 (0.6)	0 (0.0)	1 (2.7)
Carfilzomib, cyclophosphamide and dexamethasone (KCd)	1 (0.6)	0 (0.0)	1 (2.7)
Daratumumab and dexamethasone (Dd)	1 (0.6)	0 (0.0)	1 (2.7)
Cyclophosphamide, anthracyclines and related substances[b] and dexamethasone	1 (0.6)	0 (0.0)	1 (2.7)
Ixazomib, lenalidomide and dexamethasone (IxaRd)	1 (0.6)	1 (0.7)	0 (0.0)
Prednisolone and nitrogen mustard analogs	1 (0.6)	0 (0.0)	1 (2.7)
**Consolidation therapy, *n* (%)**			
Yes	2 (1.2)	2 (1.5)	0 (0.0)
No	159 (93.0)	122 (91.0)	37 (100.0)
Missing data	10 (5.8)	10 (7.5)	0 (0.0)
**Maintenance therapy, *n* (%)**			
Yes	92 (53.8)	83 (61.9)	9 (24.3)
No	68 (39.8)	40 (29.9)	28 (75.7)
Missing data	11 (6.4)	11 (8.2)	0 (0.0)
**Treatment used**			
Valid, *N (%)*[c]	92 (100)	83 (100)	9 (100)
Lenalidomide, *n* (%)	74 (80.4)	69 (83.1)	5 (55.6)
Other combinations[d], *n* (%)	18 (19.6)	14 (16.9)	4 (44.4)

2L second-line, *3L* third-line, *ATC* Anatomical Therapeutic Chemical.
[a]First documented treatment scheme (as documented by study participants); [b]ATC code: L01DB; [c]Number of patients with maintenance therapy = "Yes"; [d]Combinations/drugs used in <5 patients, including bortezomib; bortezomib and donor lymphocyte infusions; daratumumab; daratumumab and lenalidomide; daratumumab, lenalidomide and dexamethasone; experimental drug not yet approved; ixazomib; ixazomib, lenalidomide and dexamethasone; and pomalidomide; pomalidomide and dexamethasone.

Supplementary figures

Supplementary Fig. 1 Treatment outcomes in 2L and 3L index therapy, by index therapy (2L/3L), including (**a**) time to next treatment[a], (**b**) progression-free survival[b] and (**c**) overall survival[c].

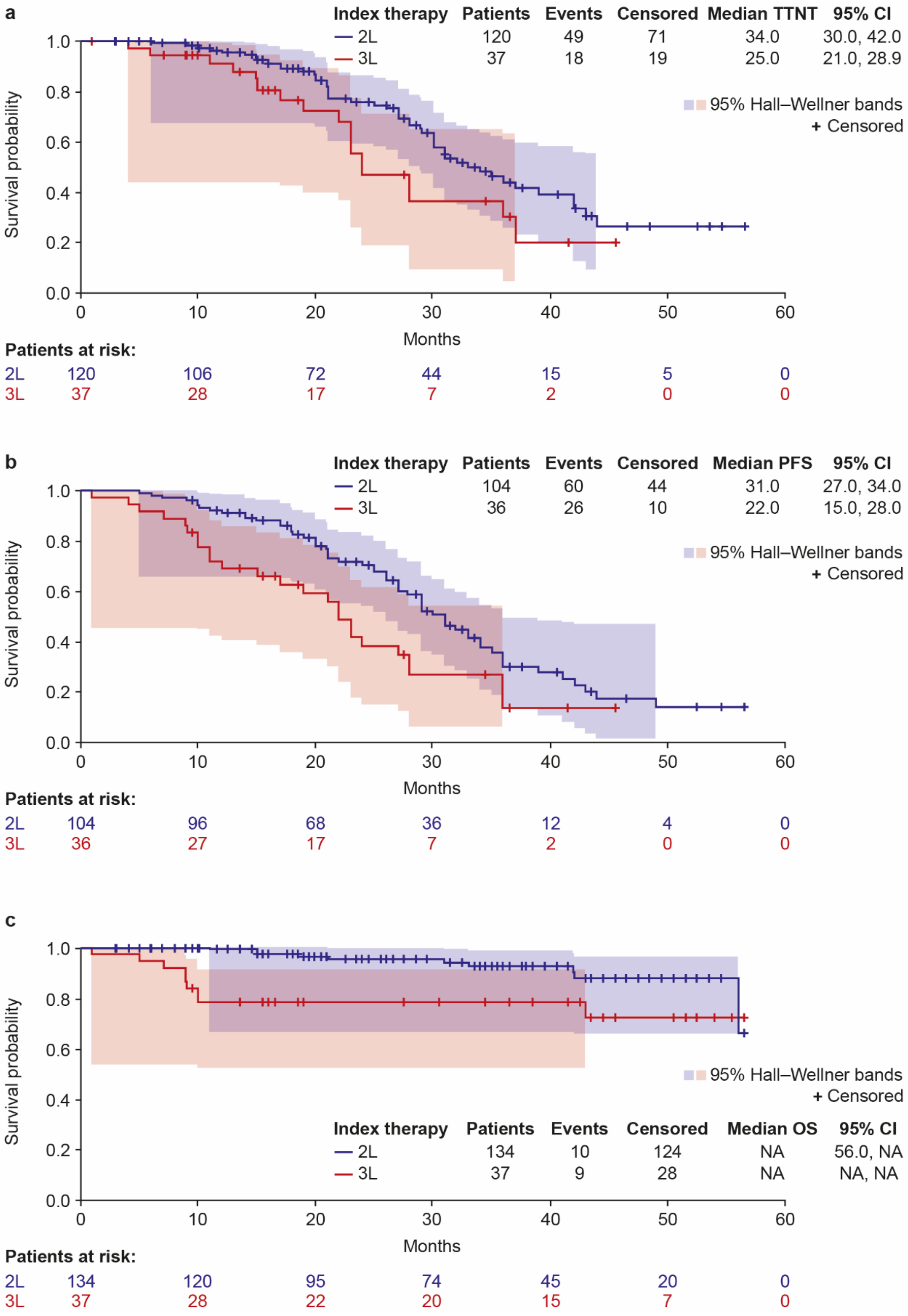

2L second-line, *3L* third-line, *CI* confidence interval, *IMWG* International Myeloma Working Group, *MM* multiple myeloma, *NA* not applicable, *OS* overall survival, *PFS* progression-free survival, *TTNT* time to next treatment.

[a]Time from the start of index therapy line to the start of next follow-up therapy line. Data from patients with no follow-up therapy lines were censored on 30 Sep 2020, date of death or date of loss to follow-up (whichever occurred first); [b]Time from the start of index therapy line to disease progression (defined by the IMWG criteria [1]), death or start of a new therapy line. Data from surviving patients without progression who were not documented as lost to follow-up were censored (for the index line) on 30 Sep 2020 if no follow-up therapy lines were documented. Patients with a new therapy line were excluded from the analysis if the start date of the new therapy line was unknown; [c]Time from the start of index therapy line to end of observation period (30 Sep 2020) or death. Patients who were alive at end of observation period (30 Sep 2020) were right censored to the end of observation period. Observation period: from the start of index therapy line to 30 Sep 2020, death or loss to follow-up (whichever occurred first).

Supplementary Fig. 2 Time to next treatment[a] in patients with and without maintenance therapy in index line.

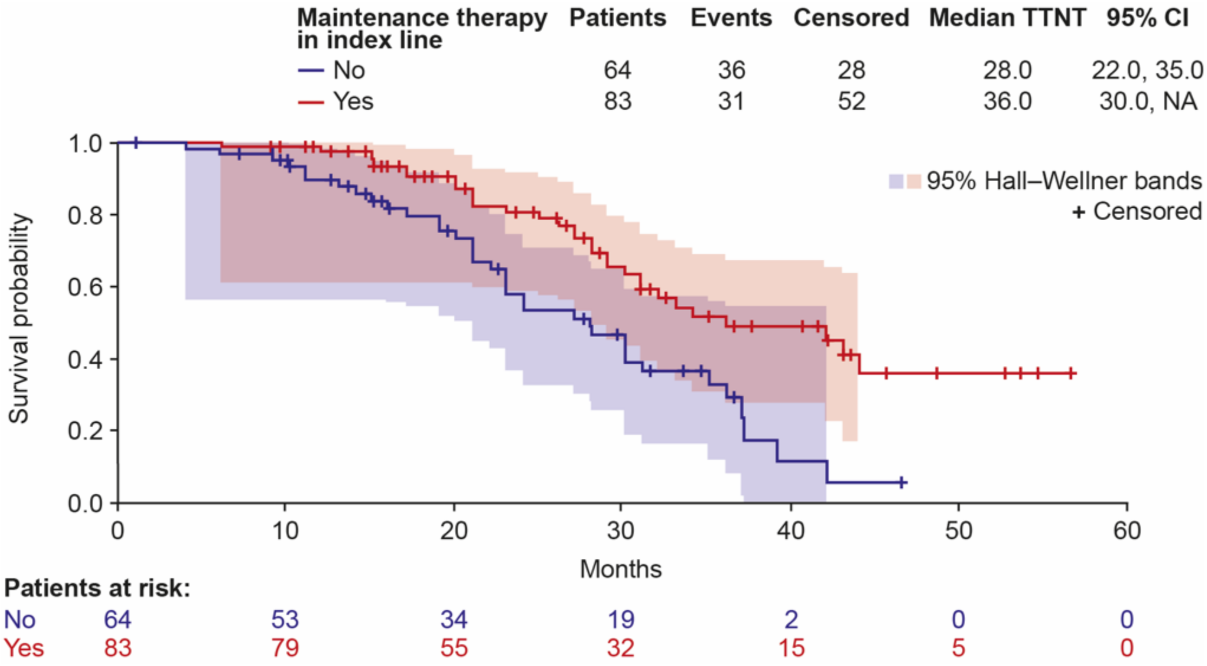

CI confidence interval, *NA* not applicable, *TTNT* time to next treatment.

[a]Time from the start of index therapy line to the start of next follow-up therapy line. Data from patients with no follow-up therapy lines were censored on 30 Sep 2020, date of death or date of loss to follow-up (whichever occurred first).

Supplementary Fig. 3 Progression-free survival[a] in patients who received carfilzomib-based and non-carfilzomib-based index therapy combinations.

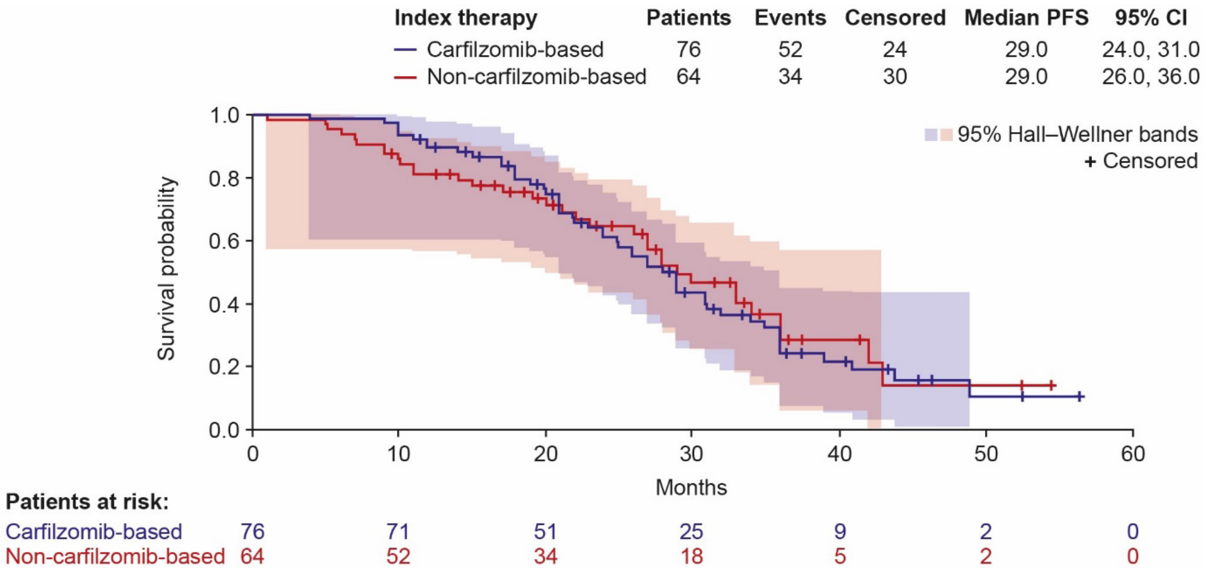

CI confidence interval, *IMWG* International Myeloma Working Group, *PFS* progression-free survival.
[a]Time from the start of index therapy line to disease progression (defined by the IMWG criteria [1]), death or start of new therapy line. Data from surviving patients without progression who were not documented as lost to follow-up were censored (for the index line) on 30 Sep 2020 if no follow-up therapy lines were documented. Patients with a new therapy line were excluded from the analysis if the start date of the new therapy line was unknown.

Supplementary reference

1. International Myeloma Working Group (IMWG). International Myeloma Working Group (IMWG) uniform response criteria for multiple myeloma. 2020. https://www.myeloma.org/resource-library/international-myeloma-working-group-imwg-uniform-response-criteria-multiple. Accessed 23 January 2024.
